# Reply to the Comments on: Tian Zhao et al. The Principle of Least Action for Reversible Thermodynamic Processes and Cycles. *Entropy* 2018, *20*, 542

**DOI:** 10.3390/e20120986

**Published:** 2018-12-18

**Authors:** Zeng-Yuan Guo, Tian Zhao, Yu-Chao Hua

**Affiliations:** Key Laboratory for Thermal Science and Power Engineering of Ministry of Education, Department of Engineering Mechanics, Tsinghua University, Beijing 100084, China

**Keywords:** principle of least action, reversible thermodynamic processes, thermodynamic cycle, Carnot cycle

## Abstract

The purpose of this reply is to provide a discussion and closure for the comment paper by Dr. Bormashenko on the present authors’ article, which discussed the application of the principle of least action in reversible thermodynamic processes and cycles. Dr. Bormashenko’s questions and misunderstandings are responded to, and the differences between the present authors’ work and Lucia’s are also presented.

Recently, the present authors noticed a comment made by Dr. Bormashenko [1] referring to the present authors’ research article which discussed the principle of least action in reversible thermodynamic processes and cycles [2]. After careful scrutiny of the comment paper, the present authors decided to provide a reply to expound the authors’ views and to bring closure to this discussion. 

1. In the comment [1], it was said that “The variational principle says nothing about the maximal time of such a movement [2,3]. Indeed, there exists an infinite number of pathways supplying the infinite time of the prescribed motion. Consequently, there exists an infinite number of thermal (*T*, *S*) pathways supplying the maximum heat to the system. Thus, the principle of least action was applied by the authors erroneously.” 

This deduction is not appropriate. The action for the brachistochrone problem is the time and the corresponding action for the thermodynamic heat absorption process is the reciprocal of heat for the fixed entropy difference between states A and B, rather than heat itself. Consequently, there exists a unique pathway supplying the maximum heat to the system, and an infinite number of pathways supplying the minimum heat to the system. Thus, there is no problem in applying the principle of least action to the thermodynamic heat absorption process.

2. In the comment [1] it was said that “Consider an arbitrary thermal engine following the thermal cycle. The maximal efficiency of the engine corresponds to the cycle, for which the ratio *Q*_2_/*Q*_1_ is minimal. Thus, the ratio *Q*_2_/*Q*_1_ should be minimized. This demand is very different from the “maximization of heat” suggested in Reference [1].”

The “maximization of heat” in the present authors’ article refers to the optimization problem of the heat absorption process with fixed entropy change, while the minimized *Q*_2_/*Q*_1_ ratio is the optimization problem of thermodynamic cycles in the comment written by Bormashenko [1]. These two problems are quite different. In addition, the brachistochrone problem is analogous to the heat absorption process, rather than the thermodynamic cycle.

3. In the comment [1], it was said that “The efficiency of the Carnot cycle has been already derived successfully with the principle of least thermodynamic action.” 

The present authors’ work is different from Lucia’s previous work [3], which was cited in the comment [2]. The major differences are (1) the principle of least action for reversible thermodynamic processes is proposed first, and the principle of least action for thermodynamic cycles is derived based on the action for thermodynamic processes; (2) the physical meaning of the action proposed in the presented authors’ work is clear and easy to follow.

4. In the comment [1], it was said that “The dimension of “thermodynamic action” suggested in Reference [1] is K^−1^, which is quite obscure and it is not defined as a mathematic functional.”

The action proposed in Lucia’s work [3] has the dimension of Joule (J), which is also different from the dimension of the action in classical mechanics and quantum mechanics, i.e., J·s. Moreover, as an example, numerous research works [4,5,6] treat the entropy generation rate as the action for heat conduction processes, whose dimension is W·K^−1^, while Hua et al. [7] proved that the entransy dissipation rate should be the action for heat conduction processes, whose dimension is W·K. Therefore, it can be seen that the dimension of the action is not fixed for different problems or for the same problem with different constraints.
